# Effect of Chemotherapy Patient Education Using the Teach-Back Method on Symptom Management and Quality of Life: A Randomized Controlled Trial

**DOI:** 10.1007/s13187-024-02564-0

**Published:** 2025-01-07

**Authors:** Belkis Gullu Gucuyener, Bilgi Gulseven Karabacak

**Affiliations:** 1 Nursing Department, Institute of Health Science, Marmara University, Basibuyuk Mah., Sureyyapasa Basibuyuk Yolu Sok., No:4/B, Pk: 34854, Maltepe, Istanbul, Turkey; 2 Nursing Department, Faculty of Health Science, Marmara University, Istanbul, Turkey

**Keywords:** Teach-back method, Chemotherapy, Quality of life, Nursing

## Abstract

This study aimed to evaluate the impact of the teach-back method in managing chemotherapy symptoms and improving quality of life. A secondary aim was to develop more effective care and education frameworks for cancer treatment. A single-center, randomized controlled trial was conducted with 80 patients who received chemotherapy between June 2022 and May 2023. Patients in the intervention group were educated about the chemotherapy process using the teach-back method, while those in the control group received standard education. Data were collected using a participant information form, the Edmonton Symptom Assessment Scale (ESAS), and the EQ-5D Quality of Life Scale. Statistical significance was accepted as *p* < 0.05 for all tests. In both groups, EQ-5D scores increased with the number of chemotherapy cycles, indicating a negative impact on quality of life. However, this increase was smaller in the intervention group. As the number of cycles increased, the intervention group scored lower on the Edmonton Symptom Assessment Scale compared to the control group. The results of the study show that using the teach-back method in patient education is effective in the management of chemotherapy-related symptoms and improving overall quality of life.

## Introduction

Cancer is one of the most important health problems afflicting societies worldwide, and its incidence and prevalence are steadily increasing. According to 2022 data from the International Agency for Research on Cancer, 19.3 million new cancer cases were diagnosed and 9.9 million people died from cancer [1]. Chemotherapy is widely used to treat cancer and involves the use of natural or synthetic chemicals, biologic agents, and hormones. Although chemotherapy drugs aim to suppress the growth, development, and enlargement of cancer cells, they often damage healthy cells as well, leading to adverse side effects [2].

Common symptoms associated with chemotherapy, such as gustatory changes, nausea and vomiting, anorexia, cachexia, pain, alopecia, and mucositis, can cause treatment delays or even discontinuation. These symptoms negatively impact the patient’s ability to participate in social and professional activities, reduce quality of life, and adversely affect survival outcomes [3].

Quality of life has been described as one’s overall satisfaction with life, influenced by one’s physical, psychological, and economic well-being, as well as the capacity to maintain positive interpersonal relationships and devote time to personal development and leisure activities. Health-related quality of life encompasses physical, emotional, functional, and social dimensions and is impacted both by the symptoms of disease and the adverse effects of treatment [4]. Improving quality of life is especially important for cancer patients [5].

Evidence-based practices are essential for symptom management in patients receiving chemotherapy [6]. Patients and their families often face challenges due to a lack of knowledge about treatment, inadequate communication with healthcare professionals, and the complexity of the healthcare system. Nurses can play a critical role in addressing these challenges [7]. By maintaining effective communication during patient care, nurses can detect symptoms early and provide appropriate care management to improve patients’ quality of life [8].

The teach-back method is a participatory communication approach in which healthcare professionals ask patients to relay back information in their own words to verify their understanding [9]. This method ensures healthcare professionals convey important information effectively by demonstrating patients’ understanding. When patients explain what they learned in their own words, healthcare professionals can assess whether the patient has understood the information correctly. In this way, the teach-back method helps clinicians identify misunderstandings and adjust their communication strategies accordingly [10].

The present study examined the effects of education delivered using the teach-back method to patients starting chemotherapy on their management of chemotherapy-related symptoms and quality of life.

## Materials and Methods

### Study Design and Setting

This randomized controlled trial was conducted between June 2022 and May 2023 at the chemotherapy center of a private hospital in Kocaeli, in western Turkiye. Permission was obtained from the authors via e-mail to use the Edmonton Symptom Assessment Scale (ESAS) and the EQ-5D Quality of Life Scale for this research. Ethical approval was obtained from the Marmara University Faculty of Medicine Clinical Research Ethics Committee (no: 09.2021.616, date: 2 July 2021). In addition, written institutional approval was obtained from the private hospital where the research was conducted. Informed consent was obtained from all patients.

The study was conducted in accordance with the Declaration of Helsinki and the Law on Medical Research Involving Human Subjects and was registered in the US Clinical Trials Registry (ID No: NCT05825287).

The study population consisted of patients diagnosed with cancer for the first time (lung, breast, gastrointestinal, genitourinary, and head and neck cancers, skin tumors, and soft tissue tumors).

### Participants and Sample

The sample size was calculated by power analysis using the G*Power (v3.1.7) program based on Cohen’s effect size coefficient [11]. To exceed 95% power with a significance level of 5% and effect size of 0.5, the necessary sample number was determined as 70 patients (35 per group). To account for potential losses during the study, we recruited a total of 80 patients, 40 in each group. All patients assigned to the intervention and control groups completed the study.

### Inclusion and Exclusion Criteria

Patients who were over 18 years of age, were receiving chemotherapy for the first time, received intravenous and oral chemotherapy, spoke Turkish, and volunteered to participate in the study were included. Patients who received radiotherapy in addition to chemotherapy or had any communication problems or cognitive impairment were excluded.

### Randomization

Patients who met the inclusion criteria were assigned to one of the groups using a random number generator (https://randomizer.org). Participants in the intervention group received patient education delivered using the teach-back method, while those in the control group received standard patient education.

### Study Variables

The independent variables of the study included age, gender, education level, occupation, income level, marital status, chronic disease status, and type of patient education received. Dependent variables were symptoms experienced (e.g., nausea, vomiting, pain, infection, diarrhea, constipation, difficulty swallowing, fatigue, hair loss, skin and nail changes, sleep disturbance, psychological problems) and quality of life.

### Data Collection Tools

#### Participant Information Form

This form was created by the researchers and consisted of a total of 18 questions including the patient’s age, gender, marital status, smoking and alcohol use, employment, education level, chronic diseases, and previous knowledge about chemotherapy.

#### Edmonton Symptom Assessment Scale

This scale, developed by Bruera et al. [12], includes items evaluating eight symptoms common among cancer patients (pain, fatigue, nausea, sadness, anxiety, sleepiness, loss of appetite, and shortness of breath), as well as an item assessing well-being and a final item for other problems that can be specified by the patient.

The validity and reliability study of the ESAS in Turkiye was carried out by Yeşilbalkan et al. [13]. Another study was conducted by Kurt and Unsar [14]. The ETS was shown to be a valid and reliable scale. Based on the literature, Kurt and Unsar added three additional symptoms often seen in patients (skin and nail changes, oral wounds, and hand numbness) to the “other problems” section of the scale. Patients rate the severity of each symptom from 0 to 10. A score of 0 indicates that the symptom is absent, and a score of 10 indicates that the symptom is at its most severe. The highest score that can be obtained from the Turkish version of the scale is 120 points.

#### EQ-5D Quality of Life Scale

The EQ-5D is a general health-related Quality Of Life Scale developed by the EuroQoL group [15]. It consists of five dimensions: mobility, self-care, pain/discomfort, anxiety/depression, and daily activities. In the 3L version, each dimension is rated on three levels, “no difficulty,” “moderate difficulty,” and “extreme difficulty,” with higher scores indicating poorer quality of life. Each of the scale dimensions yields a score ranging from − 0.59 to 1. Negative values indicate states such as immobility and unconsciousness, a score of 0 indicates death, and a value of 1 indicates perfect health. The scale also includes a visual analog scale (VAS) on which the individual rates their perceived general health status between 0 and 100. The scale was found to be valid and reliable for use in Turkiye by Kahyaoğlu Süt and Ünsar [16].

### Data Collection

In hospitals, educational materials are generally used to explain the adverse effects that patients receiving chemotherapy treatment may experience and methods of coping with them. In this study, a “Chemotherapy Education Booklet” based on the teach-back method was prepared by the researchers to be used in the training of the intervention group. The booklet was presented to 14 experts and statistically evaluated with the DISCERN scale. The DISCERN total score average was found to be 69.07, indicating the quality of the booklet was excellent. The control group was provided education using “The A-to-Z Chemotherapy Guide”, which was updated in 2021 by the institution’s Patient Education Board in March 2021 and is currently used for standard patient education in the hospital. After both groups received patient education, data were obtained by administering the ESAS and EQ-5D Quality of Life Scale. The results were analyzed to compare the effectiveness of the education.

The first session of patient education was held 30 min before receiving chemotherapy for the first time. Education sessions were repeated in both the intervention and control groups on days 14, 21, and 28, on which they were scheduled to receive chemotherapy. All sessions were conducted by the researcher in individual face-to-face meetings held in the patient education room, and each education session was also attended by the chemotherapy case manager. Education sessions lasted 20 min on average for patients in the control group and 30 min on average for those in the intervention group.

On the fifth day after each education/chemotherapy session (on days 6, 19, 26, and 33), patients were contacted by phone to administer the EQ-5D and ESAS again.

**Fig. 1:**
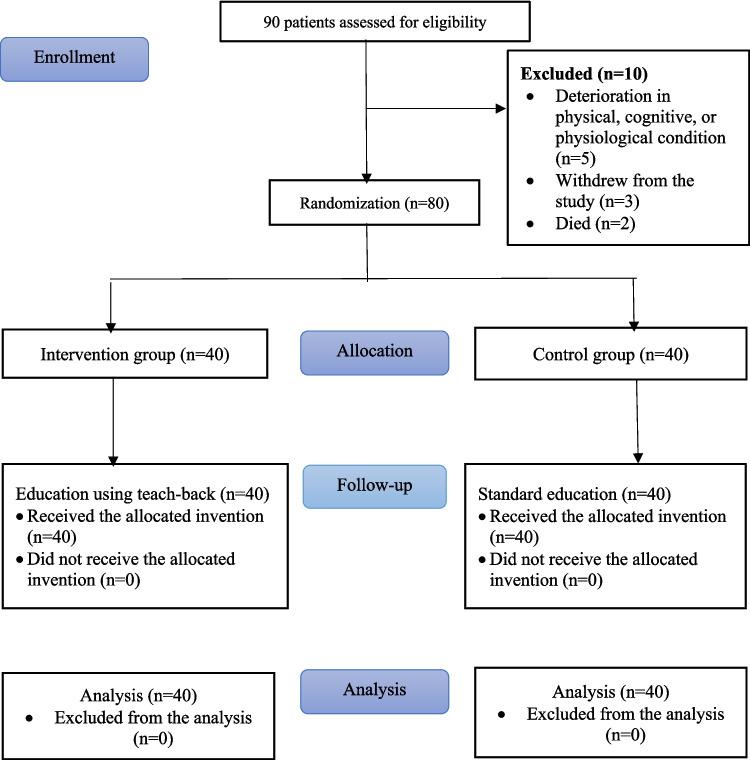
CONSORT flow diagram of the study

Table 1 shows the stages of the research, including data collection, education, and counseling. The CONSORT flow chart of the study is given in Fig. 1.

**Table 1:** Data collection, education, and counseling performed in the study

Obtain study approval (ethics committee/institutional approval) Randomization of intervention and control groups	
Intervention group (*n* = 40)	Control group (*n* = 40)
**Chemotherapy initial education**	**Chemotherapy initial education**
• Provide information about the study and obtain informed consent • Complete the participant information form • Administer the Edmonton Symptom Assessment Scale (ESAS) • Administer the EQ-5D Quality of Life Scale (EQ-5D) • Provide education about chemotherapy • Ask the patient to explain what they learned using the teach-back method	• Provide information about the study and obtain informed consent • Complete the participant information form • Administer the Edmonton Symptom Assessment Scale (ESAS) • Administer the EQ-5D Quality of Life Scale (EQ-5D) • Provide education about chemotherapy
**Phone call on day 5 after chemotherapy cycle 1**	**Phone call on day 5 after chemotherapy cycle 1**
• Administer the ESAS and EQ-5D • Provide education and counseling as needed by the patient	• Administer the ESAS and EQ-5D • Provide education and counseling as needed by the patient
**Chemotherapy cycle 2**	**Chemotherapy cycle 2**
• Repeat the education using the teach-back method and provide counseling • Ask the patient to provide post-education feedback via a structured form	• Repeat the training given with the standard method and provide counseling
**Phone call on day 5 after chemotherapy cycle 2**	**Phone call on day 5 after chemotherapy cycle 2**
• Administer the ESAS and EQ-5D • Provide education and counseling as needed by the patient	• Administer the ESAS and EQ-5D • Provide education and counseling as needed by the patient
**Chemotherapy cycle 3**	**Chemotherapy cycle 3**
• Repeat the education using the teach-back method and provide counseling • Ask the patient to provide post-education feedback via a structured form	Repeat the training given with the standard method and provide counseling
**Phone call on day 5 after chemotherapy cycle 3**	**Phone call on day 5 after chemotherapy cycle 3**
• Administer the ESAS and EQ-5D • Provide education and counseling as needed by the patient	• Administer the ESAS and EQ-5D • Provide education and counseling as needed by the patient
**Chemotherapy Cycle 4**	**Chemotherapy cycle 4**
• Repeat the education using the teach-back method and provide counseling • Ask the patient to provide post-education feedback via a structured form	Repeat the training given with the standard method and provide counseling
**Phone call on day 5 after chemotherapy cycle 4**	**Phone call on day 5 after chemotherapy cycle 4**
• Administer the ESAS and EQ-5D • Provide education and counseling as needed by the patient	• Administer the ESAS and EQ-5D • Provide education and counseling as needed by the patient

### Outcomes

The primary outcomes of the study were symptom management and quality of life after chemotherapy.

### Blinding

Double blinding was not possible in this study because the researcher applied the intervention. However, since the participants did not know their group assignment, single-blinding was achieved. Moreover, data analysis was performed by an external biostatistics specialist, resulting in blinding during the analysis phase.

### Statistical Analysis

The demographic and clinical characteristics of the patients in the study were summarized using number and frequency values. ESAS and EQ-5D scores were summarized using mean and standard deviation. Parametric tests were used for statistical analysis because there were more than 30 participants in each group [17–19]. The groups were compared with the chi-square test, Fisher’s test, and independent-samples *t*-test. Comparisons of the data within groups were performed using repeated measures analysis of variance (ANOVA). For this test, the Greenhouse–Geisser correction was applied if the results of Mauchly’s test of sphericity indicated non-homogeneous variances. The paired-samples *t*-test with the Bonferroni correction was used for multiple comparisons of variables that differed within a group. Statistical significance was accepted at *p* < 0.05 in all analyses. The data were analyzed using the IBM SPSS Statistics version 27 statistical package program.

## Results

Table 2 presents the comparative analysis of the control and intervention groups according to their descriptive characteristics. A majority of participants in the intervention group were aged 45–54, while the 55–64 age group was the largest in the control group (*p* < 0.05). Moreover, participants in the intervention group had higher education levels than those in the control group (*p* < 0.001).

**Table 2:** Comparison of participants’ descriptive characteristics by group (*N* = 80)

Characteristic	Intervention group (*n* = 40)	Control group (*n* = 40)	Chi-square statistic	*p*
**Age (years)**				
35–44	8 (72.7%)	3 (27.3%)	10.354	**0.035**
45–54	12 (66.7%)	6 (33.3%)		
55–64	10 (37%)	17 (63%)		
65–74	4 (26.7%)	11 (73.3%)		
75–84	6 (66.7%)	3 (33.3%)		
**Weight (kg)**				
40–49	0 (0%)	3 (100%)	5.039	0.539
50–59	4 (36.4%)	7 (63.6%)		
60–69	12 (52.2%)	11 (47.8%)		
70–79	14 (58.3%)	10 (41.7%)		
80–89	5 (55.6%)	4 (44.4%)		
90–99	2 (40%)	3 (60%)		
100–109	3 (60%)	2 (40%)		
**Gender**				
Female	28 (50.91%)	27 (49.09%)	0.058	0.809
Male	12 (48.00%)	13 (52.00%)		
**Marital status**				
Single	7 (53.85%)	6 (46.15%)	0.092	0.762
Married	33 (49.25%)	34 (50.75%)		
**Education level**				
Elementary school	1 (50.00%)	1 (50.00%)	38.500	** < 0.001**
Middle school	0 (0.00%)	10 (100.00%)		
High school	4 (16.00%)	21 (84.00%)		
University and higher	35 (81.40%)	8 (18.60%)		
**Employment status**				
Employed	19 (52.78%)	17 (47.22%)	0.202	0.653
Not employed	21 (47.73%)	23 (52.27%)		
**Occupation**				
Government employee	8 (72.73%)	3 (27.27%)	-	**0.015**
Private sector employee	8 (47.06%)	9 (52.94%)		
Self-employed	4 (33.33%)	8 (66.67%)		
Homemaker	0 (0.00%)	7 (100.00%)		
Other	20 (60.61%)	13 (39.39%)		
**Caregiver**				
Yes	22 (37.29%)	37 (62.71%)	14.500	** < 0.001**
No	18 (85.71%)	3 (14.29%)		
**Smoking**				
Yes	4 (66.67%)	2 (33.33%)	-	0.675
No	36 (48.65%)	38 (51.35%)		
**Alcohol use**				
None	8 (25.81%)	23 (74.19%)	11.800	** < 0.001**
On special occasions	32 (65.31%)	17 (34.69%)		

*n*, number of patients; %, row percentage; *p*, statistical significance

Statistically significant results are indicated by bold font to highlight their significance

Table 3 shows the comparative analysis of clinical data between the control and intervention groups. The only significant difference between the groups was that 12 participants in the intervention group but none in the control group were receiving any long-term treatment other than for diabetes or hypertension (*p* < 0.001).

**Table 3:** Comparison of participants’ clinical characteristics by group (*N* = 80)

Characteristic	Intervention group (*n* = 40)	Control group (*n* = 40)	Chi-square statistic	*p*
**Other long-term treatment**				
Yes	12 (100.00%)	0 (0.00%)	14.100	** < 0.001**
No	28 (41.18%)	40 (58.82%)		
**Comorbidity**				
Diabetes mellitus (DM)	6	6	2.220	0.528
Hypertension (HT)	4	6		
DM + HT	2	5		
**Regular medication use**				
Yes	12 (60.00%)	8 (40.00%)	1.070	0.302
No	28 (46.67%)	32 (53.33%)		
**Chemotherapy knowledge**				
Yes	11 (37.93%)	18 (62.07%)	2.650	0.104
No	29 (56.86%)	22 (43.14%)		
**Family history of chemotherapy**				
Yes	17 (54.84%)	14 (45.16%)	0.474	0.491
No	23 (46.94%)	26 (53.06%)		
**Accompanied a family member receiving chemotherapy**				
Yes	3 (42.9%)	4 (57.1%)	0.157	0.692
No	37 (50.7%)	36 (49.3%)		
**Number of chemotherapy cycles**				
4	21 (52.5%)	19 (47.5%)	2.567	0.699
6	14 (46.7%)	16 (53.3%)		
8	1 (33.3%)	2 (66.7%)		
10	1 (50%)	1 (50%)		
12	3 (75%)	1 (25%)		
14	0 (0%)	1 (100%)		
**Grade**				
1	1 (2.5%)	2 (5%)	0.939	0.625
2	29 (72.5%)	31 (77.5%)		
3	10 (25%)	7 (17.5%)		
**Cycle frequency**				
Every 14 days	13 (56.5%)	10 (43.5%)	1.412	0.494
Every 21 days	24 (50%)	24 (50%)		
Every 28 days	3 (33.3%)	6 (66.7%)		
**Cancer type**				
Lung	8 (42.1%)	11 (57.9%)	5.786	0.448
Breast	24 (60%)	16 (40%)		
Gastrointestinal system	4 (30.8%)	9 (69.2%)		
Genitourinary system	1 (50%)	2 (66.7%)		
Head and neck	1 (50%)	1 (50%)		
Skin	1 (100%)	0 (0%)		
Soft tissue	1 (50%)	1 (50%)		

*n*, number of patients; %, row percentage; *p*, statistical significance

Statistically significant results are indicated by bold font to highlight their significance

Table 4 shows the distribution of chemotherapeutic agents received by the participants. There was no statistically significant difference between the groups in terms of the chemotherapy agents’ mechanisms of action (*p* > 0.05).

**Table 4:** Comparison of chemotherapeutic agents received by group (*N* = 80)

Pharmacological mechanism/feature	Intervention group (*n* = 40)	Control group (*n* = 40)	Chi-square statistic	*p*
**Use of targeted therapy**				
Yes	4 (10%)	3 (7.5%)	0.157	0.692
No	36 (90%)	37 (92.5%)		
**Use of platinum derivatives**				
Yes	19 (47.5%)	12 (30%)	2.580	0.108
No	21 (52.5%)	28 (70%)		
**Use of topoisomerase inhibitors**				
Yes	0 (0.0%)	1 (2.5%)	1.400	0.237
No	40 (100%)	39 (97.5%)		
**Use of microtubule inhibitors**				
Yes	23 (57.5%)	22 (55%)	0.0508	0.822
No	17 (42.5%)	18 (45%)		
**Use of antimetabolites**				
Yes	12 (30.0%)	13 (32.5%)	0.0582	0.809
No	28 (70.0%)	27 (67.5%)		
**Use of alkylating agents**				
Yes	24 (60.0%)	18 (45.0%)	1.800	0.179
No	16 (40.0%)	22 (55.0%)		
**Use of anti-tumor antibiotic**				
Yes	3 (7.5%)	6 (15%)	1.150	0.284
No	37 (92.5%)	34 (85%)		

*n*, number of patients; %, row percentage; *p*, statistical significance

Table 5 shows the comparison of ESAS scores of the participants in the control and intervention groups before the first chemotherapy cycle and 5 days after each cycle. ESAS scores were statistically similar before the first cycle but significantly lower in the intervention group after each cycle (*p* < 0.001 for all).

**Table 5:** Comparison of Edmonton Symptom Assessment Scale scores of participants in the control and intervention groups measured at different times (*N* = 80)

Time	Subscales	Intervention group (*n* = 40)	Control group (*n* = 40)	*t*	*p*
		Mean ± SD	Mean ± SD		
	*Pain*	0 ± 0	0 ± 0	-	-
	*Fatigue*	0 ± 0	0 ± 0	-	-
	*Nausea*	0.050 ± 0.316	0 ± 0	1.000	0.323
	*Sadness*	0 ± 0	0 ± 0	-	-
	*Anxiety*	0 ± 0	0 ± 0	-	-
	*Insomnia*	0 ± 0	0 ± 0	-	-
**Before cycle 1**	*Loss of appetite*	0 ± 0	0 ± 0	-	-
	*Well-being*	0 ± 0	0 ± 0	-	-
	*Shortness of breath*	0.025 ± 0.158	0 ± 0	1.000	0.323
	*Skin and nail changes*	0 ± 0	0 ± 0	-	-
	*Mouth sores*	0 ± 0	0 ± 0	-	-
	*Hand numbness*	0 ± 0	0 ± 0	-	-
	*Other*	0 ± 0	0 ± 0	-	-
	*Pain*	1.325 ± 1.328	3.075 ± 1.421	− 5.690	** < 0.001**
**After cycle 1**	*Fatigue*	5.675 ± 1.163	5.800 ± 1.244	− 0.464	0.644
	*Nausea*	3.900 ± 1.128	4.125 ± 1.522	− 0.751	0.455
	*Sadness*	0 ± 0	1.075 ± 1.185	− 5.737	** < 0.001**
	*Anxiety*	1.050 ± 1.197	1.750 ± 1.193	− 2.619	**0.051**
	*Insomnia*	0.300 ± 0.687	0.950 ± 1.358	− 2.702	**0.009**
	*Loss of appetite*	3.975 ± 1.349	4.600 ± 1.566	− 1.913	0.059
	*Well-being*	2.275 ± 1.485	3.450 ± 1.300	− 3.766	** < 0.001**
	*Shortness of breath*	0.550 ± 0.846	0.575 ± 1.130	− 0.112	0.911
	*Skin and nail changes*	1.975 ± 1.609	2.250 ± 1.932	− 0.692	0.491
	*Mouth sores*	0.775 ± 0.891	0.775 ± 1.349	0.000	1.000
	*Hand numbness*	1.650 ± 1.075	2.200 ± 2.301	− 1.370	0.176
	*Other*	2.167 ± 0.707	2.318 ± 1.129	− 0.517	**0.608**
	*Pain*	0.850 ± 1.051	3.850 ± 1.511	− 10.305	** < 0.001**
	*Fatigue*	4.450 ± 0.959	5.725 ± 1.301	− 4.989	** < 0.001**
	*Nausea*	2.975 ± 0.92	4.675 ± 1.474	− 6.188	** < 0.001**
	*Sadness*	0 ± 0	0.850 ± 1.231	− 4.367	** < 0.001**
	*Anxiety*	0.875 ± 0.966	2.150 ± 1.075	− 5.579	** < 0.001**
	*Insomnia*	0.425 ± 0.931	1.150 ± 1.210	− 3.004	**0.004**
**After cycle 2**	*Loss of appetite*	3.225 ± 1.000	5.275 ± 1.396	− 7.552	** < 0.001**
	*Well-being*	1.825 ± 1.010	3.400 ± 1.374	− 5.842	** < 0.001**
	*Shortness of breath*	0.400 ± 0.778	0.725 ± 1.219	− 1.421	0.160
	*Skin and nail changes*	1.725 ± 1.467	2.775 ± 1.928	− 2.741	**0.008**
	*Mouth sores*	0.775 ± 1.025	0.875 ± 1.488	− 0.350	0.727
	*Hand numbness*	2.375 ± 1.628	3.075 ± 2.368	− 1.540	0.128
	*Other*	2.167 ± 0.707	2.174 ± 1.072	− 0.025	0.980
	*Pain*	0.775 ± 1.074	3.525 ± 1.519	− 9.350	** < 0.001**
	*Fatigue*	3.800 ± 0.853	5.4 ± 1.128	− 7.155	** < 0.001**
	*Nausea*	2.775 ± 0.920	4.625 ± 0.952	− 8.838	** < 0.001**
	*Sadness*	0 ± 0	0.95 ± 1.26	− 4.769	** < 0.001**
	*Anxiety*	0.675 ± 0.888	1.9 ± 1.236	− 5.089	** < 0.001**
	*Insomnia*	0.150 ± 0.580	0.95 ± 1.339	− 3.468	**0.001**
**After cycle 3**	*Loss of appetite*	2.900 ± 0.928	4.3 ± 1.604	− 4.777	** < 0.001**
	*Well-being*	1.325 ± 0.971	3.35 ± 1.312	− 7.848	** < 0.001**
	*Shortness of breath*	0.333 ± 0.662	0.675 ± 1.248	− 1.525	0.133
	*Skin and nail changes*	1.825 ± 1.412	2.35 ± 1.916	− 1.395	0.167
	*Mouth sores*	0.575 ± 0.813	0.8 ± 1.344	− 0.906	0.368
	*Hand numbness*	2.200 ± 1.418	2.3 ± 2.028	− 0.256	0.799
	*Other*	2.167 ± 0.707	2.174 ± 1.072	− 0.025	0.980
	*Pain*	0.825 ± 1.152	2.975 ± 1.31	− 7.793	** < 0.001**
	*Fatigue*	3.725 ± 1.198	5.125 ± 1.324	− 4.959	** < 0.001**
	*Nausea*	2.625 ± 0.868	4.05 ± 1.037	− 6.667	** < 0.001**
	*Sadness*	0 ± 0	1.25 ± 1.498	− 5.278	** < 0.001**
	*Anxiety*	0.650 ± 0.921	1.775 ± 1.291	− 4.487	** < 0.001**
	*Insomnia*	0.050 ± 0.221	1.025 ± 1.405	− 4.336	** < 0.001**
**After cycle 4**	*Loss of appetite*	2.850 ± 1.189	4.025 ± 1.609	− 3.715	** < 0.001**
	*Well-being*	1.125 ± 1.017	3.45 ± 1.377	− 8.590	** < 0.001**
	*Shortness of breath*	0.325 ± 0.694	0.575 ± 1.13	− 1.193	0.237
	*Skin and nail changes*	1.600 ± 1.429	2.525 ± 1.948	− 2.422	**0.058**
	*Mouth sores*	0.550 ± 0.783	0.825 ± 1.375	− 1.099	0.276
	*Hand numbness*	2.250 ± 1.515	2.3 ± 2.278	− 0.116	0.908
	*Other*	2.167 ± 0.707	2.174 ± 1.072	− 0.025	0.980
*TOTAL (before cycle 1)*		0.075 ± 0.474	0 ± 0	1.000	0.323
*TOTAL (5 days after cycle 1)*		23.450 ± 5.32	30.625 ± 7.164	− 5.086	** < 0.001**
*TOTAL (5 days after cycle 2)*		19.900 ± 4.413	34.525 ± 6.312	− 12.009	** < 0.001**
*TOTAL (5 days after cycle 3)*		17.325 ± 4.817	31.125 ± 5.739	− 11.649	** < 0.001**
*TOTAL (5 days after cycle 4)*		16.575 ± 4.094	29.9 ± 7.414	− 9.951	** < 0.001**

*t*, independent-samples *t* test; *p*, significance value

Statistically significant results are indicated by bold font to highlight their significance

Table 6 shows the results of the comparison of the EQ-5D quality of life and VAS general health scores of the participants in the intervention and control groups. EQ-5D scores were significantly higher (indicating lower quality of life) in the control group after the second, third, and fourth cycles compared to the intervention group (*p* < 0.001). VAS scores for overall health were higher in the control group before the first chemotherapy cycle but were significantly higher in the intervention group after the second, third, and fourth cycles (*p* < 0.001 for all).

**Table 6:** Comparison of EQ-5D Quality of Life Scale and VAS scores of participants in the control and intervention groups (*N* = 80)

Time	Measurement time	Intervention group (*n* = 40)	Control group (*n* = 40)	Intergroup comparisons	
		Mean ± SD	Mean ± SD	*t*	*p*
		0.081 ± 0	0.081 ± 0	0.000	1.000
	Day 5 after cycle 1 (b)	0.154 ± 0.082	0.170 ± 0.063	− 0.936	0.352
	Day 5 after cycle 2 (c)	0.118 ± 0.063	0.598 ± 0.193	− 14.948	** < 0.001**
	Day 5 after cycle 3 (d)	0.130 ± 0.079	0.698 ± 0.185	− 17.858	** < 0.001**
	Day 5 after cycle 4 (e)	0.163 ± 0.106	0.793 ± 0.136	− 23.065	** < 0.001**
***EQ-5D* (*intragroup comparison*)**		*F* = 7.824	*F* = 210.500		
		***p* < 0.001 (a < b,c,d,e)**	***p* < 0.001 (a < b,c,d,e and b < c,d,e and c < e)**		
**VAS score**	Before cycle 1 (a)	81.75 ± 10.595	91.75 ± 11.522	− 4.041	** < 0.001**
	Day 5 after cycle 1 (b)	88.75 ± 8.224	84.75 ± 13.202	1.626	0.109
	Day 5 after cycle 2 (c)	90.5 ± 7.828	77.25 ± 11.091	6.173	** < 0.001**
	Day 5 after cycle 3 (d)	90.5 ± 7.828	70.75 ± 8.286	10.958	** < 0.001**
	Day 5 after cycle 4 (e)	82.5 ± 7.425	67.25 ± 7.506	9.135	** < 0.001**
**VAS score (*intragroup comparison*)**		*F* = 19.259	*F* = 107.793		
		***p* < 0.001 (a < b,c,d,e and e < b,c,d)**	***p* < 0.001 (a > b,c,d,e and b > c,d,e)**		

*F*, repeated measures ANOVA; *t*, independent-samples *t* test; *p*, significance value

Statistically significant results are indicated by bold font to highlight their significance

## Discussion

The teach-back method is an approach designed to improve individuals’ understanding of the information conveyed in education sessions by asking them to repeat the key points of the information [20]. This method allows educators to verify that patients have understood and are able to explain basic information as they understand it. When patients repeat the information back to the educator in their own words, it confirms their understanding. In addition, this technique can help health educators identify explanations and communication strategies that are frequently misunderstood by patients [21].

The United States Agency for Healthcare Research and Quality, which is tasked with improving the safety and quality of American healthcare infrastructure, developed a module on implementing the teach-back methodology. The module aims to enable healthcare professionals to convey information in a way that patients can understand and to provide the necessary support to patients and their families throughout the diagnosis and treatment process [22].

In this study, the participants in the intervention group were younger and more educated than the control group (Table 2). However, the two groups were homogeneous in terms of other demographic characteristics, clinical characteristics, chemotherapeutic agents used, and symptoms and quality of life before chemotherapy. This is important when interpreting the effectiveness of education delivered with the teach-back method.

As there was no statistical difference in the distribution of chemotherapy agents received by participants in the intervention and control groups (*p* > 0.05) (Table 4), we assume the effects of the drugs on quality of life were similar in both groups.

Camara et al. compared changes in quality of life caused by adjuvant anthracycline/taxane chemotherapy with and without gemcitabine in a sample of 3691 women with high-risk breast cancer. Their results showed that long-term outcomes were similar between the two groups [23].

In a randomized controlled trial involving 1581 patients, Moehler et al. observed a more pronounced improvement in quality of life among patients with gastric/gastroesophageal junction cancer and esophageal adenocarcinoma receiving a combination of first-line nivolumab and chemotherapy compared to those treated with chemotherapy alone [24]. Compared with the current literature, our findings are consistent with some studies but not with others. However, it was not possible to analyze the impact of different chemotherapy agent regimens on quality of life due to the inclusion of a small sample of patients with various types of cancer.

In our study, there was no difference in the ESAS scores of the intervention and control groups before the first cycle of chemotherapy (*p* > 0.05). However, ESAS scores were higher for participants in the intervention group after all chemotherapy cycles when compared with the control group (*p* < 0.05) (Table 5). This shows that the intervention group experienced fewer symptoms.

In a study by Maleki et al. aiming to increase medication adherence among patients with head and neck cancer receiving radiotherapy, a pharmaceutical care program was compared to standard care in the radiotherapy clinic. They utilized the teach-back technique as an educational approach and assessed patients using the Medication Understanding and Use Self-Efficacy scale as well as the ESAS. The findings showed that ESAS scores at discharge were significantly lower in patients who received education using the teach-back method [25].

A systematic review by Choi and Choi including four quasi-experimental studies and one randomized controlled trial with cancer patients demonstrated that implementing the teach-back method in intervention programs for cancer patients had a significant impact on health literacy, symptom experience, death anxiety, self-efficacy, happiness, and distress [26].

The results of our research support those of leading studies in the field and indicate that the teach-back method offers a more effective educational paradigm than traditional educational methods.

In this study, a decline in quality of life was observed in both groups after starting chemotherapy (Table 5). This shows that chemotherapy has negative consequences on individuals and significantly impairs their quality of life. In a randomized controlled study involving 120 participants, Osama et al. reported significant differences in functional status, symptomatology, and overall quality of life of individuals with breast and colon cancer between assessments conducted before and after their first chemotherapy cycle (*p* < 0.001). The patients had higher physical, role, emotional, cognitive, and social functioning, fewer symptoms, less financial burden, and better general health before compared to after first-cycle chemotherapy [27]. Our findings are consistent with other studies in the literature.

Comparisons of quality-of-life scores revealed no difference between the groups before and after the first cycle, whereas the intervention group reported higher quality of life than the control group starting after the second cycle. In addition, when the groups’ VAS scores for perceived overall health were compared, we noted that participants in the control group reported significantly better overall health than those in the intervention group. This may be related to the fact that none of the patients in the control group required long-term treatment for other conditions. However, the participants in the control group exhibited a significantly greater decrease in VAS score after second, third, and fourth chemotherapy cycles compared to the participants in the intervention group (Table 5). This suggested that the education provided to patients using the teach-back method was more effective than the standard education received by the control group.

In a prospective quasi-experimental study conducted by Peng et al. involving 88 participants, researchers evaluated the perioperative pain severity VAS scores of lung cancer patients and determined that nursing interventions using the teach-back method were associated with lower scores than standard educational practices [28]. Consistent with the literature, our results demonstrate that incorporating the teach-back method into patient education programs can reduce pain and improve well-being.

## Conclusion

Patient education provided using the teach-back method was shown to improve chemotherapy patients’ treatment-related symptom management and quality of life. These educational interventions help patients cope more effectively with the physical and emotional challenges that arise during treatment.

Translating these findings into clinical practice may offer an important opportunity for the development of more effective patient support programs in the field of cancer treatment. The results of this study could be a valuable guide for healthcare professionals in providing better support and care to cancer patients throughout their treatment process.

## Study Strengths

During the data collection process, all participants received treatment and education individually and therefore could not influence one another. In addition, education was provided to all participants by the same researcher.

## Study Limitations

A limitation of this study is that the sample group did not consist of patients with the same cancer type or receiving the same chemotherapy drugs. Although the study was originally planned to be conducted with gastric, colon, and pancreatic cancer patients, those with other cancers were also included in the study due to a decline in the number of patient admissions to the hospital where the study was conducted resulting from bureaucratic factors. However, the lack of a statistical difference between the groups in terms of medical diagnosis and cancer type suggests that this limitation is acceptable.

## Data Availability

The datasets used and/or analyzed during the current study can be obtained from the first author upon reasonable request.

## Code Availability

No software or custom code was used in the study.
